# Modern Approaches in the Identification and Quantification of Immunogenic Peptides in Cereals by LC-MS/MS

**DOI:** 10.3389/fpls.2019.01470

**Published:** 2019-11-14

**Authors:** Thais O. Alves, Carolina T. S. D’Almeida, Katharina A. Scherf, Mariana S. L. Ferreira

**Affiliations:** ^1^Food and Nutrition Graduate Program (PPGAN), Laboratory of Bioactives, Federal University of the State of Rio de Janeiro (UNIRIO), Rio de Janeiro, Brazil; ^2^Laboratory of Protein Biochemistry—Center of Innovation in Mass Spectrometry (LBP-IMasS), UNIRIO, Rio de Janeiro, Brazil; ^3^Department of Bioactive and Functional Food Chemistry, Institute of Applied Biosciences, Karlsruhe Institute of Technology (KIT), Karlsruhe, Germany

**Keywords:** allergenic peptides, cereals, gluten, LC coupled to mass spectrometry, multiple reaction monitoring, prolamins, proteomics

## Abstract

Celiac disease (CD) is an immunogenic disorder that affects the small intestine. It is caused by the ingestion of gluten, a protein network formed by prolamins and glutelins from cereals such as wheat, barley, rye and, possibly, oats. For predisposed people, gluten presents epitopes able to stimulate T-cells causing symptoms like nausea, vomiting, diarrhea, among others unrelated to the gastrointestinal system. The only treatment for CD is to maintain a gluten-free diet, not exceeding 20 mg/kg of gluten, what is generally considered the safe amount for celiacs. Due to this context, it is very important to identify and quantify the gluten content of food products. ELISA is the most commonly used method to detect gluten traces in food. However, by detecting only prolamins, the results of ELISA tests may be underestimated. For this reason, more reliable and sensitive assays are needed to improve gluten quantification. Because of high sensitivity and the ability to detect even trace amounts of peptides in complex matrices, the most promising approaches to verify the presence of gluten peptides in food are non-immunological techniques, like liquid chromatography coupled to mass spectrometry. Different methodologies using this approach have been developed and described in the last years, ranging from non-targeted and exploratory analysis to targeted and specific methods depending on the purpose of interest. Non-targeted analyses aim to define the proteomic profile of the sample, while targeted analyses allow the search for specific peptides, making it possible to quantify them. This review aims to gather and summarize the main proteomic techniques used in the identification and quantitation of gluten peptides related to CD-activity and gluten-related allergies.

## Introduction

Cereals are one of the main food sources in the world. The nutrients provided by this group represent about 50% of the recommended daily intake (RDI) of carbohydrates and one third of the RDI for proteins. Cereal grains are also considered a good source of minerals and vitamins, especially complex B vitamins (Belitz et al., 2009). According to updated FAO data (2018), the cereal production, including non-food uses specially for maize, in the last year exceeded 2,600 million tons, with a slight decrease in production expected for 2019.

Wheat is one of the most important cereals in the world for human consumption, and is considered the most suitable raw material for bread and pasta making. Its production has remained constant over the years, currently only behind maize and followed by rice (FAOSTAT, 2018). In recent data reported by USDA (2018), world wheat production reached 733 million tons, whereas the estimated consumption is about 745 million tons. Barley, rye, and oats also have large production and consumption, but not so expressive as wheat, their production corresponds to about 25% of that of wheat. Rye is mostly applied for baking, while barley is applied in beer production and oats essentially commercialized as flour, bran, and other products for immediate consumption (Owusu-Apenten, 2002).

The search for practical ways in the preparation and consumption of meals combined with the promotion of healthier eating habits, sparked an increase in research for new processes for products (Pfeifer et al., 2014). Grain processing involves techniques that can alter protein structure, causing changes in solubility, viscoelastic properties, spatial conformation of proteins, and other changes (Hayta and Alpaslan, 2001). Among the main treatments used in cereal processing, extrusion and cooking can be highlighted, as well as baking and pasta production. However, there is a lack of studies to elucidate how processing may alter not only technological characteristics, but also nutritional and health implications, since cereal proteins, especially wheat, have a high allergenic potential in susceptible individuals.

The allergenic potential of cereals has been mainly related to gluten, a complex mixture of storage proteins found in cereals that is composed mainly of prolamins (responsible for the cohesiveness and extensibility of the gluten) and glutelins (maintenance of the elasticity and strength of the gluten). Gluten proteins have common structural characteristics. Their primary structure is subdivided into distinct domains that may exhibit repetitive sequences rich in the amino acids proline (P) and glutamine (Q) (Shewry and Halford, 2002), but low in amino acids with charged side groups. Different compositions in amino acids can be responsible for different reactivity associated with celiac disease (CD) (Belitz et al., 2009; Colgrave et al., 2015). Grains belonging to the *Triticeae* subtribe (wheat, barley, and rye) contain significantly higher levels of Q and P, being the main cereal grains responsible for triggering the immune response in celiacs (Colgrave et al., 2015). Cysteines represent only 2% of the amino acids of gluten proteins, but are extremely important for their structure and functionality, since they allow the formation of disulfide bonds, responsible for gluten polymerization (Wieser, 2007).

The disorders associated to gluten consumption are known as GRD (gluten-related diseases) and are classified into three types according to the response triggered in the body: autoimmune, allergic, and neither autoimmune nor allergic (Sapone et al., 2012). Examples of autoimmune diseases are dermatitis herpetiformis, gluten-induced ataxia, and CD. Among IgE antibody-mediated allergies, WDEIA (wheat-dependent exercise-induced anaphylaxis), contact urticaria, food allergy, and respiratory allergies are prominent. The respiratory allergies are related to the proteins of the albumin and globulin fractions, and are known as “baker’s asthma” (Weiss et al., 1997). There are also disorders of non-allergic and non-autoimmune origin known as non-celiac gluten sensitivity or intolerance (Sapone et al., 2012).

In all cases of GRD, diagnosed patients cannot consume foods containing gluten or its traces, since even minimal amounts can trigger the reaction, causing variable symptoms, ranging from abdominal pain, bloating, and diarrhea, to osteoporosis and long-term infertility. The severity of the reaction is due to the degree of intolerance of each individual (Pietzak and Fasano, 2005; Banerjee, 2010). Therefore, it is extremely important to correctly identify the presence of immunogenic proteins in cereal products, in order to guarantee the safety of their consumption by the patients. One major problem for patients are the “hidden sources of gluten” that may be present in foods due to inadequate labeling or cross contamination during manufacturing or transportation. There is also concern about the presence of gluten due to the tendency of its incorporation into foods that traditionally do not contain wheat in its composition (*e.g.* sausages, nuggets, meatballs) (Day et al., 2006).

Some authors indicated the natural genetic variability as a strategy to be further exploited for the development of wheat varieties with lower levels of immunogenic epitopes (Spaenij–Dekking et al., 2005). By using the R5-based quantitation of immunodominant toxic epitopes as the trait of interest, Ribeiro et al. (2016) demonstrated that tetraploid varieties had a lower amount of toxic epitopes than hexaploid varieties, especially when compared to *Triticum aestivum* landraces, which were not subjected to breeding practices. Despite the advances in the study of genetic variability of wheat toxicity, at present there is no common hexaploid wheat that might be safe for CD patients. Furthermore, considering the wide range of *in vivo* immunoresponse between celiac patients and the limitation of the immunological techniques for quantifying gluten proteins, the quantification and identification of cereal reactive proteins and peptides has been a complex task requiring constant analytical improvements.

Currently, the gold standard method to detect and quantify gluten in foods is the R5 ELISA and it is recommended by the Codex Alimentarius Commission (2008). More recently, the G12 ELISA was accepted by AOAC International as an official method of analysis, first action (Halbmayr-Jech et al., 2015). ELISAs are based on the immune reaction between specific antibodies that have been raised to detect the antigen to be determined, such as gluten. Due to their sensitivity, adequate recovery, repeatability, and reproducibility as demonstrated by collaborative studies, ELISAs are most commonly used to check for the presence of gluten in gluten-free raw materials and products. However, in some cases, ELISAs may give false negative results, because the monoclonal antibodies have been raised against prolamins (R5: raised against a rye extract and G12: raised against the α-gliadin 33-mer peptide) and are not suitable for all gluten protein types. As a consequence, the quantification can be compromised since the result is converted to gluten amount by multiplying the prolamin content by two, assuming the prolamin/glutelin ratio to be constant (Thompson and Méndez, 2008; Wieser and Koehler, 2009). ELISA methods currently cannot distinguish between the different gluten-containing cereals and are affected by the cross-reactivity of antibodies (Wieser and Koehler, 2009; Diaz-Amigo and Popping, 2013; Martínez-Esteso et al., 2017).

In this context, proteomic approaches appear to be more sensitive and reliable techniques than the currently used assays to identify gluten proteins, which present high amino acid sequence similarity and are difficult to distinguish. Especially when applying modern *in tandem* tools, proteomics can undoubtedly provide additional information to ELISA results, such as the confirmation of specific proteins by unraveling the peptide sequences (Martínez-Esteso et al., 2017).

**Figure 1:**
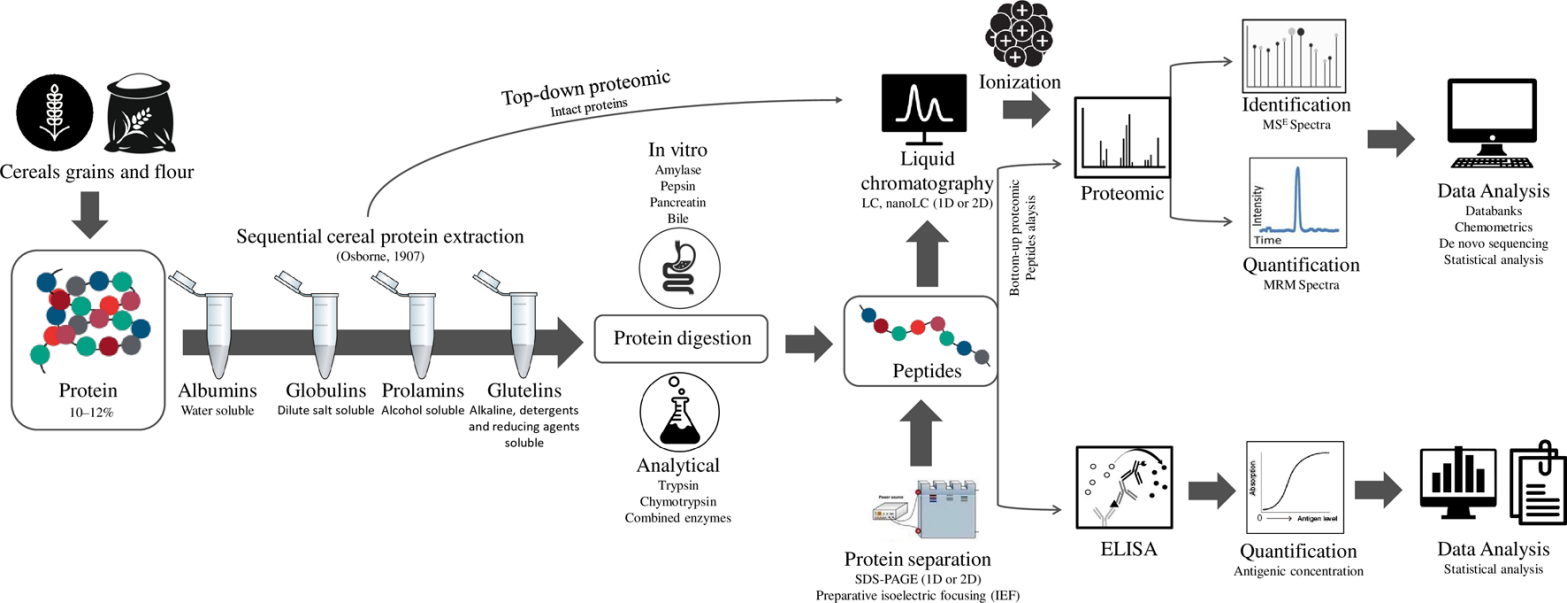
General workflow for cereal proteomic analysis.

A general workflow for cereal proteomics, as shown in **Figure 1**, should first consider the appropriate extraction taking into account the solubility of gluten proteins (Osborne, 1907) that usually requires the use of reducing (*e.g.* DTT—dithiothreitol, DTE—dithioerythritol, and TCEP-Tris2-Carboxyethyl phosphine hydrochloride) and denaturing agents (*e.g.* SDS or urea) (Schalk et al., 2018a; Schalk et al., 2018b). The enzymatic digestion is the crucial step in bottom-up proteomics. This high-throughput analysis is based on the detection of peptides to assign the proteins. The digestion is important, because the sensitivity of methods depends on the optimal size of peptides, considering the ability to be ionized and fragmented. Trypsin is the most commonly used enzyme due to its specific cleavage on the C-terminal side of lysine and arginine residues. However, due to the small number of these proteolytic cleavage sites in gluten proteins, a multiple enzymatic digestion or less specific enzymes have been used for cereal proteomics (Vensel et al., 2011; Fiedler et al., 2014). After that, the peptides can be separated by electrophoresis or liquid chromatography (LC).

LC coupled to mass spectrometry (LC-MS) is the most important tool for the identification and quantification of immunoreactive cereal proteins (Alves et al., 2017). One of the major contributions of proteomics in the study of CD has been the identification of the immunogenic epitope sequences of gluten peptides. The application of LC-MS methods makes it possible to identify the cereal species, the protein subunit, and to quantify thousands of peptides and proteins in the same experiment. Having a well-curated database that includes all possible proteins present in that organism is a great advantage for the identification of the sequences. However, peptide sequences may also be identified by *de novo* sequencing (Ferreira et al., 2014).

Other aspects, such as ionization source and type of MS analyzer, also influence the analysis and consequently the identification and quantification of the proteins. All of these topics will be briefly covered in this review. With the use of this information, significant advances in the understanding of GRD mechanisms, such as aspects related to resistance to proteolysis of these proteins and influence of cereal processing can be clarified, contributing to various aspects from the development of peptide detection and quantification methods to the selection of less reactive genotypes for better tolerability of these cereals.

## Available Gluten Protein and Customized Databases

For LC-MS/MS analysis is important to define and use a well-curated gluten protein sequence database to improve the identification of immunogenic peptides. For this, it may be necessary to build a custom database based on an existing general database.

To provide the scientific community with a high quality protein knowledge base, the Swiss Institute of Bioinformatics (SIB), the European Bioinformatics Institute (EBI), and the Protein Information Resource (PIR) group have joined forces and created the UniProt consortium in 2002 (www.uniprot.org/). The UniProt Knowledgebase (UniProtKB), the main product of this consortium, combines UniProtKB/Swiss-Prot (contains over 560,823 sequences that have been created by experimental information extracted from the literature, organized and summarized, 379 belonging to *Triticum aestivum*—accessed Oct. 2019) and UniProtKB/TrEMBL (171,501,488 sequences that have been largely derived from high throughput DNA sequencing, 142,558 belong to wheat) (The UniProt Consortium, 2017). Besides this, the UniProt consortium also produces and maintains UniRef (which consists of clusters of sequences sharing 100%, 90%, or 50% of identity), UniParc (a highly redundant archive that contains original protein sequences retrieved from several different sources), or UniMES (a collection of metagenomic and environmental sequences) (Schneider et al., 2009). All known sequences can be BLAST searched against the entire database or a part of it and the resulting sequence of high homology can be downloaded from UniProt in FASTA format.

To customize a database, other softwares should be applied. Clustal Omega (Goujon et al., 2010) and Jalview (Waterhouse et al., 2009) are used in multiple sequence alignments. Clustal Omega is an online software tool that allows protein sequences to be entered in a text file format, with optional output formats (msf output format). Jalview is a desktop program or online software for editing, visualizing, and analyzing multiple sequence alignments using Clustal Omega. Lastly, it is necessary to count the number of sequences within the file and remove redundant sequences with DBtoolkit software (Martens et al., 2005). A custom database (GluPro V1.0) of wheat gluten proteins containing 630 unique protein sequences was created to be used in LC-MS/MS data analysis to identify the presence of immunoreactive gluten peptides in foods (Bromilow et al., 2017a). All software tools mentioned above were used to create this database and it provides more reliable protein IDs compared to the general database (*Viridiplantae*).

Juhász et al. (2015) also collected datasets from various public databases (UniprotKB, IEDB, NCBI GenBank) to create a specific database addressed to cereal prolamin protein families. The ProPepper database contains 2,484 unique and complete prolamin sequences, but also their peptides obtained with single- and multi-enzyme *in silico* digestions and specific epitopes that are responsible for wheat-related food disorders. Accordingly, is provided 667,402 unique digestion events, but also including redundant protein–peptide connections due to the simultaneous presence of some protein sequences in many genotypes and the frequency of the same peptide within a protein. Besides to be highly specific in the identification of protein sequences, this database provides specific information, such as the possible disease associated with the sequence.

Developed in 2005, Allergen Online database provides a updated peer reviewed allergen list and sequence searchable dataset to offer a risk assessment tool for evaluating the potential allergenicity of new food proteins produced by genetically modified organisms (GMO) and novel protein ingredients in processed foods (Goodman et al., 2016). The main goal is identify proteins that may present a potential risk of allergenic cross-reactivity. This database currently presents a list of 72 proteins known to induce CD together with a downloadable list containing more than 1,000 CD-active peptide sequences. However, this function cannot be used to search mass spectrometry (MS) data directly due to the restrictive size and not adapted format of the database (*e.g.* not available in FASTA format).

## Proteomics as a Tool for the Screening for Immunogenic Peptides

The “omic” suffix means collectively considering all constituents. Proteomics consists of the analysis of the set of proteins encoded by the genome and its component molecules responsible for the control of almost all biological processes (Graves and Haystead, 2002). The use of proteomics in food analysis has become a key technological tool for the characterization and quantification of proteins and peptides, especially when it comes to the evaluation of biological markers (Carr and Anderson, 2008; Herrero et al., 2012). The coupling of the chromatographic separation and mass spectrometer detection techniques (LC-MS) increases the speed of the analyses, allowing a large number of samples to be analyzed in a short period of time (Alves et al., 2017). In these studies, the amount of data generated is enormous and requires an important computational analytical effort to process data in a systemic and comparative way in order to deliver a practical conclusion and application (Victorio et al., 2018).

MS analyses can be divided into two types: untargeted and targeted approaches. While untargeted approaches aim to establish a comprehensive profile of the proteome of the sample, the targeted analysis allows the selection of specific molecules to be screened and studied in the sample (Saghatelian and Cravatt, 2005). Both types follow a standard workflow, where the sample is ionized *via* an ion source; the ions are separated according to their mass-to-charge ratios (*m/z*) and monitored by a mass analyzer prior to detection. *In tandem* MS (MS/MS) these precursor ions are then introduced into a collision cell where they undergo specific fragmentation through collision-induced dissociation (CID) by an inert gas, usually nitrogen or argon, resulting in the formation of product ions (Lovric, 2011). MS/MS is usually applied for complex samples, where identified peptides are selected and subjected to fragmentation to decipher the amino acid sequence, allowing the identification of sequences that differ from each other by a single amino acid (Graves and Haystead, 2002).

The ionization source significantly impacts MS analysis as there are many ionization techniques and each has its advantages and ideal applications. The selection of the ideal ionization technique should be made based on the structure of the analyte of interest as well as the desired application (Buse et al., 2014). Various ionization techniques have been used with MS, including Electrospray Ionization (ESI), Atmospheric Pressure Chemical Ionization (APCI), Atmospheric Pressure Photoionization (APPI), and Matrix-Assisted Laser Desorption Ionization (MALDI). For the ion source, it is important to be efficient, but at the same time sensitive and “soft”, to avoid the destruction of the analyte by unwanted fragmentation in-source (Lovric, 2011). Of these, the techniques most commonly used for having this feature are ESI and MALDI (El-Aneed et al., 2009). MALDI ionization essentially generates monocharged ions and thus does not require any deconvolution step. This technique emerged as an alternative to characterize wheat storage proteins due to its robustness and ability to ionize intact proteins and tolerate the presence of contaminants, such as detergents (SDS) commonly used for gluten extraction (Ferreira et al., 2014). However, this technique cannot be hyphenated directly to LC.

Conversely, ESI is powerful technique for the analysis of complex protein and peptide mixtures that benefit from the additional separation. Jira and Münch (2019) used LC-ESI-MS/MS for the simultaneous MS detection of the six most important grain species (barley, maize, oats, rice, rye, and wheat) in meat products based on marker peptides. ESI was also suitable to detect traces of immunogenic gluten marker peptides in a variety of foods (Sealey-Voyksner et al., 2010) and gluten marker peptides (e.g., Manfredi et al., 2015; Colgrave et al., 2016; Schalk et al., 2018a; Schalk et al., 2018b).

A miniaturized version of ESI, termed nanospray, has become the preferred method of introducing large peptides into the mass spectrometer in case peptide contents are suspected to be low to very low (Nadler et al., 2017; Hopper et al., 2019). nanoLC-ESI-MS/MS was efficient to identify 29 immunogenic peptides from wheat flour carrying a high number of epitopes (Alves et al., 2018). Droplets produced from nanoESI are smaller than in conventional ESI (of the order of a few hundred nanometers), greatly improving the sensitivity and explaining the predominance of this technique in quantitative large-scale proteomics. The use of nanoLC to analyze complex peptide mixtures, especially when combined to orthogonal separation such as 2D RP/RP separation prior to MS/MS analysis, improves the resolution facilitating the identification and quantification of peptides containing CD immunogenic epitopes even at low femtomolar levels of detection (van den Broeck et al., 2015). When sample amounts are limited, nanoLC remains the best option due to the increased analytical sensitivity, otherwise UPLC or even HPLC separation is also useful for gluten detection.

Quadrupole is one of the most common type of mass analyzer, which four parallel metal rods are opposite connected electrically and voltage is applied to the diagonally placed pair of rods, resulting in an electrical field that causes the ions to travel forward. Nonetheless, a set of mass analyzers can be used for this purpose, such as ToF (Time of Flight), IT (ion trap), Orbitrap^®^, or FT-ICR (Fourier transform ion cyclotron resonance), they can also be combined to improve the sensitivity of the method (Herrero et al., 2012).

### MS-Based Identification of Immunogenic Peptides

The variability of cereal protein composition caused by the different species and varieties (genetic variability) and by growing conditions (environmental variability) leads to methodological difficulties for the analysis of immunoreactive peptides and also for the selection of genotypes (Juhász et al., 2015). In addition, the high amount of repetitive units and the similarity of the amino acid sequences of the different prolamins, with limitations in the available methodologies, make it difficult to accurately identify peptides that cause diseases related to cereal consumption, as well as their genotype frequency, variability, and stability (Juhász et al., 2015).

As mentioned, MS is considered to be the golden standard for the analysis of biomolecules in complex samples, such as food matrices, because it presents high levels of sensitivity and specificity, and has been increasingly used in food analysis (Colgrave, 2017). In cereal proteins, multiple acquisition methods or DIA (data independent acquisition), such as MS^E^ allow minimizing data loss (*e.g.*, non-fragmented precursors) (Victorio et al., 2018). In MS^E^ methods, all ions generated in the source are transmitted to the collision chamber, which alternates between low and high energy, sending precursors and fragments *quasi*-simultaneously to the TOF (Time of Flight) analyzer (Egertson et al., 2015). In DIA methods there is no previous selection of precursors or a threshold of ion intensity to undergo fragmentation, while for DDA typically the three most intensive single or multiple charged ions eluting from the column are selected for fragmentation (van den Broeck et al., 2015).

The use of label-free acquisition methods, such as the multiplex MS^E^ method, takes advantage of a data collection approach that focuses on maximizing peptide fragmentation and then improving the identification and proteome coverage (Victorio et al., 2018). MS^E^ methods have been applied to gluten protein identification and quantitation (Uvackova et al., 2013;van den Broeck et al., 2015; Bromilow et al., 2017b). Label-free absolute quantification is based on the relationship between MS signal response and protein peptide concentration: the average MS signal response for the three most intense tryptic peptides per mole of protein (top 3) is constant (CV < 10%) and this relationship is used to calculate a universal signal response factor given an internal standard (Silva et al., 2006). However, due to data complexity many steps of data processing are required in DIA such as peak alignment, ion detection, clustering, and normalization prior to peptide matching by search algorithms from a database of protein sequences.

In general, there are two possible approaches when applying LC-MS/MS for gluten detection, both of which are valid, but depend on the question to be answered. The first option is to specifically detect known CD-immunogenic peptides in order to estimate the immunogenicity of gluten. This has been reported for a selection of α- and γ-gliadin peptides (Sealey-Voyksner et al., 2010), α-gliadin peptides (van den Broeck et al., 2015), the 33-mer peptide (Schalk et al., 2017), and various gluten-derived peptides (Alves et al., 2018;Malalgoda et al., 2018). In contrast, the second option is to look for the presence of gluten, but not necessarily for CD-immunogenic peptides. Due to their length of at least nine amino acids, the poor enzymatic digestibility of the corresponding repetitive sequences, and their high contents of glutamine and proline, CD-immunogenic peptides often have properties unfavorable for MS detection, whereas other gluten peptides might be more abundant. With the overall aim to detect gluten, this approach was also used to identify marker peptides in wheat, rye, barley, and oats (Manfredi et al., 2015; Schalk et al., 2018a; Schalk et al., 2018b).

Recent examples demonstrating the successful application of proteomics in the evaluation of the presence of gluten marker peptides, include the detection of the presence of gluten in beers (Tanner et al., 2013; Allred et al., 2014). Tanner et al. (2013) also made a comparison between two different gluten detection methods, reinforcing the superiority of LC-MS/MS to detect gluten peptides in relation to the ELISA due to its higher sensibility and the ability to detect both, glutelin and prolamins, and not only prolamins as ELISA. This fact can be corroborated by Colgrave et al. (2014), where MS was used to detect and confirm the presence of hydrolyzed gluten proteins in beers which had been previously estimated as gluten-free by ELISA. A set of barley-specific peptide markers was also proposed to evaluate the contamination of processed food, ensuring the food safety for CD patients (Colgrave et al., 2016).

In fact, MS has been effectively applied to define a set of specific analytical targets, such as signature peptides specific to prolamins or cereal-containing gluten proteins. The main interest of these works is to apply new methodologies that can overcome food adulteration and mislabeling or to check authenticity of cereal based-products (**Table 1**). Bönick et al. (2017) reported an analytical strategy, based on *in silico* steps and LC-MS/MS, to check the authenticity of wheat, spelt, and rye addition in bread products. MS has been reported as a promising alternative to ELISA, in particular for the detection but also quantification of proteins in contaminated food, as it can target multiple and very specific analytes (Martínez-Esteso et al., 2016).

**Table 1:** Overview of studies using LC-MS to detect gluten in foods.

Title	Food matrix	Techniques/methods	Reference
Novel aspects of quantitation of immunogenic wheat gluten peptides by liquid chromatography–mass spectrometry/mass spectrometry	Quinoa flour; whole grain corn flour; whole grain soy flour; vital wheat gluten flour; whole wheat flour; rye flour; barley flour; rice flour; oat flour; powdered ice tea mix; pasta; orzo; cheerios; hot sauce; bread; goldfish crackers; white vinegar; toothpaste; body lotion; body wash; beer; gin; vodka; rum; red wine; white wine and GF product	HPLC-ESI-TQS-MS/MS	Sealey-Voyksner et al. (2010)
Assessment of allergenicity of diploid and hexaploid wheat genotypes: identification of allergens in the albumin/globulin fraction	Wheat; human sera	ELISA; SDS-PAGE; immunoblotting; LC-MS/MS	Larre et al. (2011)
Measuring hordein (gluten) in beer—a comparison of ELISA and mass spectrometry	Beer	Western blot; ELISA sandwich; MRM-MS	Tanner et al. (2013)
MS^E^ based multiplex protein analysis quantified important allergenic proteins and detected relevant peptides carrying known epitopes in wheat grain extracts	Wheat	NanoUPLC-QTOF-MS/MS	Uvackova et al. (2013)
The MS^E^-proteomic analysis of gliadins and glutenins in wheat grain identifies and quantifies proteins associated with celiac disease and baker’s asthma	Wheat	NanoUPLC-QTOF-MS/MS	Uvackova et al., 2013
Evaluation of qualitative and quantitative immunoassays to detect barley contamination in gluten-free beer with confirmation using LC-MS/MS	Barley; GF beer	EZ Gluten assay; AllerTek Gluten ELISA; LC-Qtof-MS/MS	Allred et al. (2014)
Characterization of grain-specific peptide markers for the detection of gluten by mass spectrometry	Gluten; wheat flour; barley flour; rye flour; oat flour	NanoHPLC-ESI-pSMR; MS/MS	Fiedler et al. (2014)
Assessment of the allergenicity of soluble fractions from GM and commercial genotypes of wheats	Wheat; GM wheat (*T. aestivum* and *T. durum*); human sera	SDS-PAGE; western blot; immunoblotting; nanoLC-Qtof-MS/MS	Lupi et al. (2014)
Specific nongluten proteins of wheat are novel target antigens in celiac disease humoral response	Wheat; Human sera	ELISA; SDS-PAGE; immunoblotting; MS/MS	Huebener et al. (2014)
Using mass spectrometry to detect hydrolysed gluten in beer that is responsible for false negatives by ELISA	Beer	ELISA; nanoHPLC-ESI-MRM-MS	Colgrave et al. (2014)
Qualitative and quantitative determination of peptides related to celiac disease in mixtures derived from different methods of simulated gastrointestinal digestion of wheat products	Durum wheat (ground kernels; semolina; dough; extruded pasta; dried pasta and cooked pasta)	LC-ESI-MS	Prandi et al. (2014)
Label free targeted detection and quantification of celiac disease immunogenic epitopes by mass spectrometry	Wheat	On-line 2D nanoLC–MS/MS; UPLC-MRM-MS/MS	van den Broeck et al. (2015)
Allergen relative abundance in several wheat varieties as revealed *via* a targeted quantitative approach using MS	Wheat (*T. aestivum, T. durum, T. monococcum*)	LC-MS/MS	Rogniaux et al. (2015)
Proteomic profiling of 16 cereal grains and the application of targeted proteomics to detect wheat contamination	Barley; wheat; rye; oats; green wheat; amaranth; chia; quinoa; sorghum; tef; buckwheat; soy; millet; maize	SDS-PAGE; western blot; nanoUPLC-ESI-MRM-MS	Colgrave et al. (2015)
Multiplex liquid chromatography–tandem mass spectrometry for the detection of wheat, oat, barley and rye prolamins towards the assessment of gluten-free product safety	Flour; seeds; pasta; biscuits; cookies; crackers; beverages; breads; breakfast cereals; snacks	HPLC-IonTrap-MS/MS	Manfredi et al. (2015)
Defining the wheat gluten peptide fingerprint *via* a discovery and targeted proteomics approach	Wheat gluten; GluVital^®^	ELISA; nanoUPLC-ESI-MS/MS	Martínez-Esteso et al. (2016)
Identification of barley-specific peptide markers that persist in processed foods and are capable of detecting barley contamination by LC-MS/MS	Barley; wheat; rye; oats; green wheat; amaranth; chia; quinoa; sorghum; tef; buckwheat; soy; millet; maize; breakfast cereals	nanoUPLC-ESI-MRM-MS	Colgrave et al. (2016)
Quantitation of the immunodominant 33-mer peptide from α-gliadin in wheat flours by liquid chromatography tandem mass spectrometry	Wheat flour	RP-HPLC; 1H qNMR; untargeted MS/MC; ESI-MRM-MS/MS	Schalk et al. (2017)
Determination of wheat, rye and spelt authenticity in bread by targeted peptide biomarkers	Wheat; spelt; emmel wheat; einkorn wheat; barley; maize; oat; rye	UPLC-ESI-MRM-MS/MS	Bönick et al. (2017)
Peptides from gluten digestion: a comparison between old and modern wheat varieties	Wheat (*T. aestivum*, *T. durum*, *T. monococcum*, *T. dicoccum*, *T. spelta*)	UPLC-ESI-MS; HPLC-ESI-MS/MS	Prandi et al. (2017)
Development and validation of the detection method for wheat and barley glutens using mass spectrometry in processed foods	Seeds; flour; beers; cookies; beverages; GF products (GF flour; corn flour; apple wine; rice wine)	ELISA; LC-ESI-MRM-MS	Liao et al. (2017)
Using LC-MS to examine the fermented food products vinegar and soy sauce for the presence of gluten	Vinegar; malt vinegar; soy sauce	ELISA; UHPLC-MRM-MS/MS	Li et al. (2018)
Differential expression of albumins and globulins of wheat flours of different technological qualities revealed by nanoUPLC-UDMS^E^	Wheat flour	nanoUPLC-HDMSE; nanoUPLC-UDMSE	Victorio et al. (2018)
Immunogenic and allergenic profile of wheat flours from different technological qualities revealed by ion mobility mass spectrometry	Wheat flour	nanoUPLC-MSE; nanoUPLC-UDMSE	Alves et al. (2018)
Detection and quantitation of immunogenic epitopes related to celiac disease in historical and modern hard red spring wheat cultivars	Wheat	RP-HPLC; SDS-PAGE; SRM-MS	Malalgoda et al. (2018)
Targeted liquid chromatography tandem mass spectrometry to quantitate wheat gluten using well-defined reference proteins	Wheat	RP-HPLC; untargeted MS/MS; MRM-MS	Schalk et al. (2018b)
Quantitation of specific barley, rye, and oat marker peptides by targeted liquid chromatography–mass spectrometry to determine gluten concentrations	Barley; rye; oat	RP-HPLC; untargeted MS/MS; MRM-MS; competitive R5-ELISA; SDS-PAGE	Schalk et al. (2018a)
A complete mass spectrometry (MS)-based peptidomic description of gluten peptides generated during *in vitro* gastrointestinal digestion of durum wheat: implication for celiac disease	Durum wheat	SDS-PAGE; UHPLC-ESI-MS/MS; UPLC-ESI-MS	Boukid et al. (2019)

Fiedler et al. (2014) identified a list of specific grain peptides of wheat, barley, rye, and oats for the detection of gluten contamination in several types of commercial flours. Specifically, targeted MS/MS method enabled the detection of two wheat peptide markers at a level of 10 ppm of wheat flour spiked into gluten-free oat flour. Martínez-Esteso et al. (2016) identified a set of unique wheat gluten peptides and proposed their use as markers of the presence of gluten related to the manifestation of CD symptoms. The authors reinforce the idea that this strategy can be applied to other allergens and that this is the first step toward the standardization of a new methodology, using LC-MS techniques, to evaluate the immunogenicity of different food matrices but also to produce reference materials, since the establishment of a set of markers is the first step to infer the presence of gluten and that enable the quantity of gluten present to be determined.

In the last decade, ion mobility spectrometry (IMS) has appeared as an analytical separation technique, especially important to the analysis of primary structures with a high degree of homology, such as gluten proteins. The IMS consists of an orthogonal separation technique, where for each value of *m/z* a spectrum of drift time is added. The drift time corresponds to the time the ion takes to cross the ionic mobility cell where an inert gas is inserted, allowing the determination of shock sections, or collision cross-section (Michaelevski et al., 2010). Thus, the ions can be further differentiated by size, shape, and charge, which allow separating by the three-dimensional conformation even peptides that present the same *m/z* or reverse peptides. In this way, the IMS can be applied to improve LC-MS and GC-MS workflows, since it increases method sensitivity by isolating the compounds of interest from background noise, improving confidence of identification, either in targeted or non-targeted approaches (Hernández-Mesa et al., 2019).

Wheat allergens from the non-gluten soluble protein fraction (albumins and globulins) have also been reported and identified by MS (Larre et al., 2011). Samples of diploid and hexaploid wheat were used to incite immunological reaction with human sera and then were subsequently analyzed and identified by MS. The analysis of 2D spots revealed by immunoblotting leads to the MS-based identification of 39 IgE-binding proteins, some of them unknown thus far as wheat allergens. A recent study evaluated albumins and globulins from different genotypes of Brazilian wheat flour through the application of MS^E^ and IMS, called UDMS^E^ (Ultra Definition Mass Spectrometry). Collectively, about 5,900 proteins and 45,000 peptides (Victorio et al., 2018) were identified in the dataset and relatively quantified with 8 peptides/protein. Alves et al. (2018) reported that some of these proteins found have been previously described and associated with the development of respiratory allergies such as baker’s asthma. Serpins, purinins, α-amylase/protease inhibitors, globulins, and farinins have also been associated with the humoral response to celiac disease (Huebener et al., 2014).

Following the same approach, Alves et al. (2018) evaluated the allergenic potential of nine wheat flours of different technological qualities by assessment of their immunogenic profiles. Peptides responsible for the manifestation of CD and other wheat-related allergies were identified in both gluten and soluble protein fractions. This work points to a relation between the variability in the expression of allergens and the technological quality of wheat flour, showing a distinct proteomic profile in flours of inferior technological quality, concluding that they can be more immunoreactive than the other qualities, especially due to the highest expression of two isoforms of serpins.

It is important to highlight that, to reach the identification of the peptide sequences by proteomic tools, the peptides must be present in the databases, so that the results obtained in the analyses can be cross-checked with those already consolidated (Altenbach et al., 2010). One of the major limitations to conducting proteomic studies in wheat was the lack of complete sequencing of the wheat genome (Bromilow et al., 2017a). It is important to note that a high percentage of non annotated proteins makes difficult the functional classification based on the basis of gene ontology. From the 414 soluble proteins found differentially expressed in common wheat flours, 85% proteins were not yet described, according to their biological function (Victorio et al., 2018). An alternative to reduce the misidentification of sequences is the use of *de novo* sequencing to assemble wheat gluten gene sequences (Zhang et al., 2014). However, recently, the complete wheat genome was released, making it possible to improve the identifications of the proteins present in this cereal, since more peptides will be annotated in the proteomic databases (Ramírez-González et al., 2018).

### MS-Based Quantification of Immunogenic Peptides

MS can also be applied for the selection and quantification of specific peptides by methods called MRM (multiple reaction monitoring) (Anderson and Hunter, 2006) or also called SRM (selected reaction monitoring) or PRM (parallel reaction monitoring) (Peterson et al., 2012), depending on the instrument manufacturer, which allow a targeted analysis of these peptides and their quantification even at minimum or trace concentrations. A set of strategies has been developed to measure the allergenic potential of various cereal species and LC-MRM/MS technology has been useful for the identification and quantification of peptides containing immunogenic epitopes at low levels of detection, such as femtomolar (van den Broeck et al., 2015). Different approaches can be used to quantify these peptides, like label-free quantification combined with external calibration.

This methodology was used by van den Broeck et al. (2015) to quantify CD immunogenic epitopes in three varieties of wheat (two hexaploid and one tetraploid). A list of nine peptides was proposed to create the calibration curves that quantified the amount of glia-α2 and glia-α20 in gluten extracts from the samples (**Table 2**). The reliability of the results depends on optimal digestion conditions and limit of detection and/or ionization properties of the peptides. Malalgoda et al. (2018) used the same approach to quantify immunogenic peptides from old and modern hard red spring wheat cultivars. Even though, it was not possible to associate the year of harvesting with the amounts of immunogenic epitopes and α-gliadin since it was randomly detected in all samples analyzed.

**Table 2:** List of gluten peptides selected for the creation of calibration curves (van den Broeck et al., 2015).

Peptide sequence
LQLQ**PFPQPQLPY**
LQLQ**PFPQPQLPYPQ**PQPF
LQLQ**PFPQPQLPYPQ**P*H*LPYPQPQPF
LQLQ**PFPQPQLPYPQPQLPYPQ**PQPF
LQLQ**PFPQPQLPYPQPQLPYPQPQLPYPQ**PQPF
**RPQQPYPQ**PQPQY
**RPQQPYPQ***S*QPQY
QQQLIPCRDVVL
QQILQQQLIPCRDVVL

CD-epitope sequences within the peptides are shown in bold

Schalk et al. (2017) developed a targeted LC-MS/MS method to quantify the immunodominant gluten peptide called 33-mer (LQLQPFPQPQLPYPQPQLPYPQPQLPYPQPQPF), which contains three different overlapping T-cell epitopes (PFPQPQLPY; PYPQPQLPY; PQPQLPYPQ) that initiate a strong immunological response (Shan et al., 2002). In this study, the quantitative data on contents of 33-mer peptide in different wheat cultivars was carried out by combining a stable isotope dilution assay with LC-MS/MS, as first reported for peptides by Stöcklin et al. (1997). The authors detected the presence of this peptide in 23 common wheat flours and in two spelt flours (*T. spelta*), but it was absent in tetraploid and diploid wheat flours. No obvious cluster formation between modern and old wheat cultivars and no correlations between contents of 33-mer and those of α-gliadins, gliadins, gluten, or crude protein were observed. Indeed, the harvest year had a higher influence on 33-mer contents than the cultivar. It is important to highlight that this was the first study that accurately quantitated the 33-mer peptide in wheat flours.

Recent studies use the combination of untargeted and targeted methods as a strategy to quantify gluten marker peptides in cereals and determine gluten concentrations in different types of samples (Schalk et al., 2018a; Schalk et al., 2018b). Schalk et al. (2018b) developed a methodology that allowed the simultaneous determination of 33 marker peptides, 16 for wheat, seven for rye, seven for barley, and three for oats using LC-MS/MS in MRM mode, using a labeled peptide as internal standard. Furthermore, they compared the LC-MS/MS results with those of R5 ELISA RP-HPLC and GP-HPLC-FLD (gel-permeation high-performance liquid chromatography with fluorescence detection) and found a strong correlation between LC-MS/MS and the other methods. When analyzing wheat starch samples, the LC-MS/MS and ELISA results agreed well in four out of seven cases, but there were two samples where LC-MS/MS found substantially higher and one with lower gluten contents than ELISA. The lower values obtained by LC-MS/MS may be explained by the presence of other gluten peptides that were not monitored with the targeted method, whereas the higher values may be due to variable gliadin/glutenin ratios in wheat starches that may lead to an underestimation of gluten contents by ELISA (Schalk et al., 2018a).

**Figure 2:**
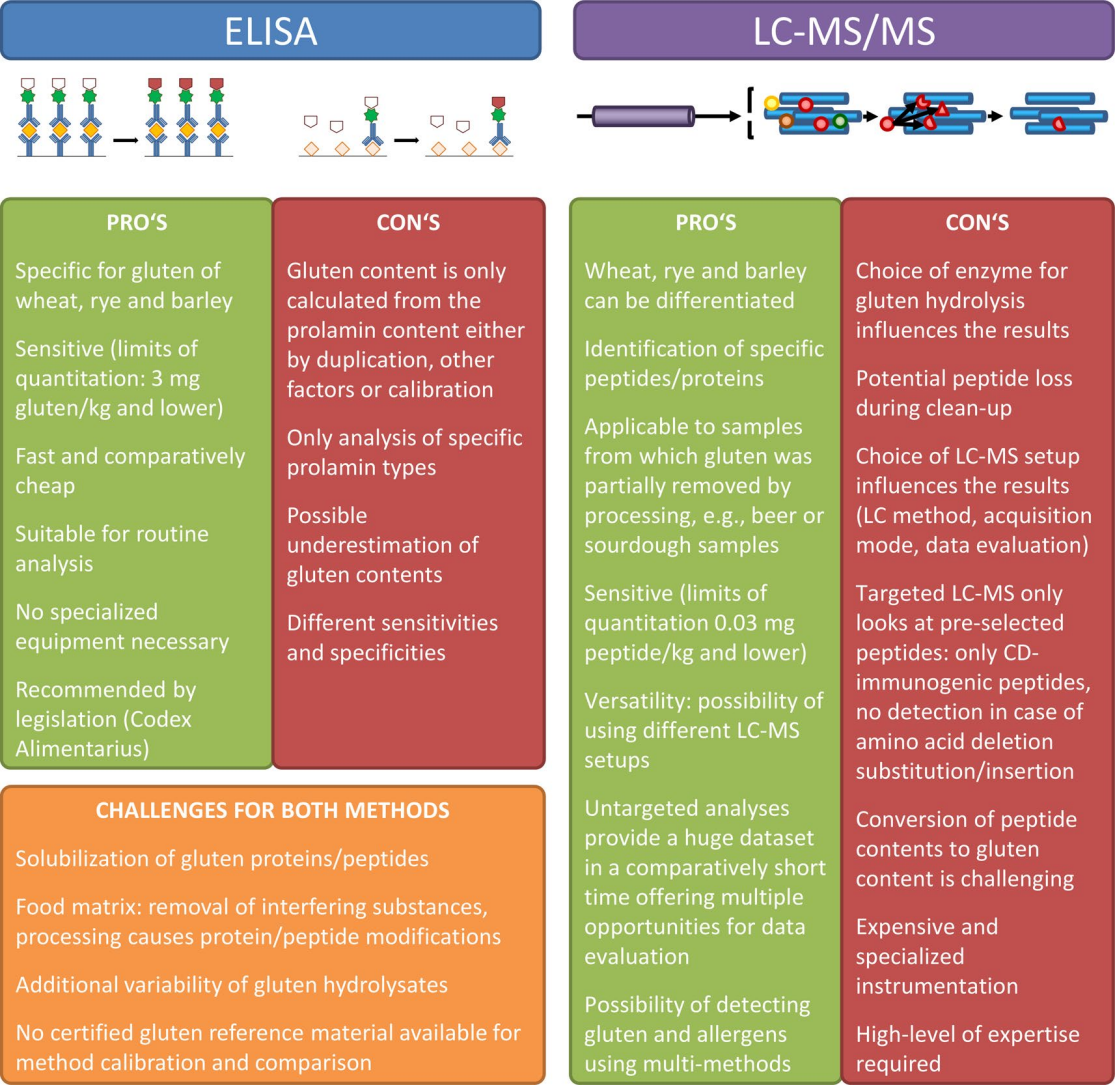
Overview of advantages and disadvantages of ELISA and LC-MS/MS for gluten detection.

One of the most important considerations when using targeted LC-MS/MS is the careful selection of gluten marker peptides, because only these pre-defined peptides will be monitored. Even a single amino acid substitution, deletion, insertion, or post-translational modification will result in that marker peptide not being detected anymore, even if the sample may still contain other gluten-derived and possibly immunogenic peptides. While it is possible to use stable isotope labeled peptides or concatamers as internal standards to precisely quantify the selected peptides, the conversion of gluten peptide contents to gluten contents is far from being trivial. Legislation requires the result to be expressed as mg gluten/kg of the food, so that the correspondence between the amount of gluten and the resulting peptides needs to be established by careful calibration, also considering the whole sample preparation procedure. One of the most important points to verify is the extent of enzymatic hydrolysis. Matrix-matched calibration has been applied in many cases (Fiedler et al., 2014; Manfredi et al., 2015), but the use of well-defined gluten reference materials revealed the complexity of converting marker peptide contents to gluten contents (Schalk et al., 2018a; Schalk et al., 2018b). Further pro’s and con’s of using ELISA and LC-MS/MS for gluten detection are given in **Figure 2**.

A quantitative approach was also used to compare the relative abundance of 12 allergens in the albumins/globulins fraction in seven wheat varieties (Rogniaux et al., 2015). Allergens were monitored by targeted investigation of one to two proteotypic peptides (single protein peptides), and the abundance of some allergens was found to be quite stable among genotypes, while others, such as α-amylase inhibitors, showed clear differences depending on the wheat species, revealing themselves as possible markers of allergenicity in wheat. The content of allergenic polypeptides from these fractions was also investigated in common and genetically modified wheat (Lupi et al., 2014) revealing a large variation in the amounts of these allergens. The lack of information on the peptide sequences and epitopes responsible for the allergies triggered by albumins/globulins render targeted studies in this protein fraction even more complicated.

## Other Strategies to Unravel and to Detect Gluten Peptides

Even with all the benefits of LC-MS/MS, such as the identification and quantification of specific proteins and peptides, new techniques have also been highlighted, such as the use of biosensors. Soler et al. (2016) used Surface Plasmon Resonance (SPR), a biosensor able to detect and quantify chemical and biological analytes quickly, sensitively, and specifically in complex field samples. SPR was able to detect gluten toxic peptides in the urine of CD patients and directly quantify the small digestive peptides without the need for prior extraction or purification procedures, so that the assay can be performed in 20 min. White et al. (2018) developed a floating gate transistor biosensor with longer analysis time (1.5 h), but it was still able to quantify wheat proteins faster than ELISA.

In addition to the shortest analysis time, biosensors also have high sensitivity at low detection limits and low cost. Chu et al. (2012) used a quartz crystal microbalance (QCM) immunosensor to detect gliadin in foods and had high sensitivity, being able to detect 8 ppb of this protein. In addition, the cost of materials for biosensor analyses is estimated to be approximately threefold less than the cost of a single ELISA kit (Soler et al., 2016). In the future, immunosensors may be promising alternatives for existing immunochemical tests, such as ELISAs, because of their specificity and sensitivity (Scherf et al., 2016). However, this method does not allow the characterization of proteins and their respective peptides, as in LC-MS/MS. In addition, the type of sensor that is the best candidate to replace the ELISA still needs to be evaluated.

LC-MRM/MS analysis can also be linked to genomics to improve our understanding of the genes responsible for expressing allergenic proteins, culminating in the development of wheat varieties with a lower allergenic potential (Salentijn et al., 2013), increasing the variety of food options that can be consumed by GRD patients by ensuring food safety. Moreover, the studies about authenticity requires also an approach towards a well-defined “proteogenomic annotation” looking carefully at the specific peptide candidates from an enzymatic digest (Bönick et al., 2017).

## Concluding Remarks and Perspectives

The use of LC-MS/MS strategies is the most useful and promising path to improve the identification and quantification of immunogenic peptides. Despite the methodological difficulties, it proves to be a fast, sensitive, and reproducible method. In addition, it can be extended to several other allergenic food matrices, like dairy, nuts, and seafood. Thus, knowing the profile of allergenic proteins of cereals is necessary as a basis, not only for future applications of MS in the quantification of gluten in food, but also to ensure the safety of consumers regarding food labeled cereal- or gluten-free.

Although the declaration of gluten-containing cereals on products labeled gluten-free is mandatory worldwide, there is no certified reference material available for gluten. The available reference material contains only gliadins that underestimate the gluten content, besides the problem of reproducing a new batch with similar properties and composition. The majority of MS-based studies have been conducted with the final objective to establish a reference material for gluten analysis starting from the study of specific grain peptide markers. Therefore, targeted high-resolution MS/MS methods allowed the quantification of low levels of specific marker peptides from different species and protein types.

When comparing LC-MS/MS methods to ELISA for gluten detection, ELISA still remains the method of choice in most cases, because it is fast, comparatively cheap, suitable for routine analyses, and does not require highly specialized equipment. However, several studies have shown that ELISA may underestimate gluten contents especially in processed foods that have been extensively heat-treated or hydrolyzed. Untargeted LC-MS/MS is recommended to screen for the presence of gluten-derived peptides in products such as beer, malt vinegar, and fermented sauces. However, there are some points that will equally all analytical methods, because gluten extractability has been shown to be reduced substantially in heat-treated foods and processing-induced post-translational protein modifications will lead to reduced gluten detectability irrespective of the analytical method used.

The use of modern MS-based techniques, combining orthogonal separations with high sensitivity and reliable certified references materials will hopefully help to better comprehend the effect of food processing or plant breeding on gluten immunogenicity. Continued efforts in this area will also help to solve the questions about the selection of relevant target epitopes and even antibodies, taking account the high protein polymorphism and the fact that patients react individually to different proteins and present variable sensitivities.

## Author Contributions

TA organized and wrote the manuscript and the summarized table, CD’A complemented the writing and designed the figure, KS reviewed the manuscript, and MF supervised and reviewed the manuscript.

## Funding

This work was funded by Federal University of State of Rio de Janeiro (UNIRIO), FAPERJ (E-26/202.709/2018), CNPq (427116/2018-0), and in part by the Coordenação de Aperfeiçoamento de Pessoal de Nível Superior—Brasil (CAPES)—Finance Code 001.

## Conflict of Interest

The authors declare that the research was conducted in the absence of any commercial or financial relationships that could be construed as a potential conflict of interest.

## Abbreviations

APCI, atmospheric pressure chemical ionization; APPI, atmospheric pressure photoionization; BLAST, Basic Local Alignment Search Tool; CCS, collision cross-section; CD, celiac disease; DIA, data independent analysis; EBI, European Bioinformatics Institute; ELISA, enzyme-linked immunosorbent assay; ESI, electrospray ionization; FT-ICR, Fourier transform ioncyclotron resonance; GMO, genetic modified organisms; GP-HPLC-FLD, high performance liquid chromatography with gel permeation and fluorescence detection; GRD, gluten related disorders; IMS, ion mobility spectrometry; IT, ion trap; MRM, multiple reaction monitoring; MS^E^, multiplex data-independent acquisition; LC-MS/MS, liquid chromatography coupled to mass spectrometry; MALDI, matrix-assisted laser desorption ionization; PIR, Protein Information Resource; PRM, parallel reaction monitoring; QMC, quartz crystal microbalance; Q, quadrupole; QqQ, triple quadrupole; SIB, Swiss Institute of Bioinformatics; SPR, surface plasmon resonance; SRM, single or selected reaction monitoring; ToF, time of flight; UDMS^E^, ultra definition mass spectrometry; WDEIA, wheat-dependent exercise-induced anaphylaxis.
